# How CEO Ethical Leadership Influences Top Management Team Creativity: Evidence From China

**DOI:** 10.3389/fpsyg.2020.00748

**Published:** 2020-05-21

**Authors:** Jinguo Zhao, Wei Sun, Shujie Zhang, Xiaohong Zhu

**Affiliations:** ^1^School of Management, Qilu University of Technology (Shandong Academy of Sciences), Jinan, China; ^2^School of Management, Shandong University, Jinan, China

**Keywords:** CEO ethical leadership, TMT cohesion, TMT creativity, power distance, top management team

## Abstract

The creative thinking and ability of top management team (TMT) members is important in coping with rapid changes in the external environment and improving the competitive advantage of an organization. This research focuses on the CEO–TMT interface to explain how CEOs influence TMT characteristics, which in turn affects TMT outcomes. Based on social learning theory, this study examines the associations among CEO ethical leadership, TMT cohesion, and TMT creativity in a Chinese context using a total of 91 TMTs. To verify the reliability and validity of the constructs, a series of confirmatory factor analyses (CFAs) were run. The results showed that the hypothetical model captured distinct constructs and fits the data well. A multistep regression method was used to test hypotheses. The results indicated that: (a) CEO ethical leadership has a positive effect on TMT creativity; (b) TMT cohesion plays a mediating role in the relationship between CEO ethical leadership and TMT creativity; and (c) power distance plays a moderating role in the relationship between CEO ethical leadership and TMT creativity. The greater the power distance, the weaker the positive relationship between CEO ethical leadership and TMT creativity. This study demonstrates the value of CEO ethical leadership and advocates the importance of establishing team cohesion and building a psychologically safe environment to motivate top managers within an organization to share information and knowledge to improve creativity.

## Introduction

In the knowledge economy, creativity plays a key role in organizational development (Shin et al., 2012; Gong, 2018). The ability of teams to effectively innovate and achieve creative output is crucial to organizations (Shepherd et al., 2017). As the decision maker of an organization, the top management team (TMT) is not only important human capital within the organization but also a core group that affects the strategic choices and business performance of the organization (Brown et al., 2005; Hoogh and Hartog, 2008; Tu and Lu, 2013; Tang et al., 2019). Therefore, TMT creativity, which is defined as the generation of novel and useful ideas for products, services, processes, and procedures by the TMT (Shin and Zhou, 2007; Shin et al., 2012), plays an important role in organizations in generating new ideas (Dewett, 2004; Lopez-Cabrales et al., 2009).

Chief executive officer (CEO) leadership is important in promoting TMT creativity (Tang et al., 2019). According to social learning theory, individuals can learn how to behave properly by observing the behaviors of role models (Bandura and Walters, 1977). Specifically, observers can learn what behavior is expected, rewarded, and punished via role modeling. In organizations, leaders are a significant and likely source of such modeling due to their assigned role, their status and success in the organization, and their power to affect the behavior and outcomes of others (Sims and Lorenzi, 1992; Brown et al., 2005). Just as Bandura (1986, p. 207) proclaimed, high standing in a “prestige hierarchy” and the ability to control rewards both contribute to modeling effectiveness.

Due to increasing attention on corporate social responsibility and business ethics, scholars and practitioners have recognized the importance of ethical leadership (e.g., Brown and Treviño, 2006; Tang et al., 2015; Chen and Hou, 2016; Tu et al., 2017). Ethical leadership refers to the demonstration of normatively appropriate conduct through personal actions and interpersonal relationships, and the promotion of such conduct to followers through two-way communication, reinforcement, and decision making (Brown et al., 2005). Ethical behavior (e.g., honesty, trustworthiness, fairness, and care) makes leaders legitimate and credible role models. Some researchers have shown that ethical leadership predicts outcomes such as perceived effectiveness of leaders, followers’ job dedication and satisfaction, and followers’ willingness to make constructive contributions to the organization (Brown et al., 2005). Furthermore, team members imitate the behavior of ethical leaders to care for and respect each other, and be more inclusive of others’ different opinions (Mayer et al., 2012). It enhances the quality of the team members’ relationship and then increases team cohesion (Barrick et al., 1998; Aubke et al., 2014), which in turn promotes team innovation (Guler and Nerkar, 2012). Based on this understanding, it can be inferred that ethical leadership can influence team members’ behavior and promote team cohesion by shaping role models, which can further stimulate team creativity. The available literature in the field of ethical leadership is somewhat theoretical, and most studies have focused on the outcomes at the individual level (Brown et al., 2005; Hoogh and Hartog, 2008; Tu and Lu, 2013). How CEO ethical leadership affects team-level results (e.g., TMT creativity) is still unclear (Feng et al., 2018). Thus, this study aims to fill these research gaps in ethical leadership from the perspective of social learning theory.

**FIGURE 1:**
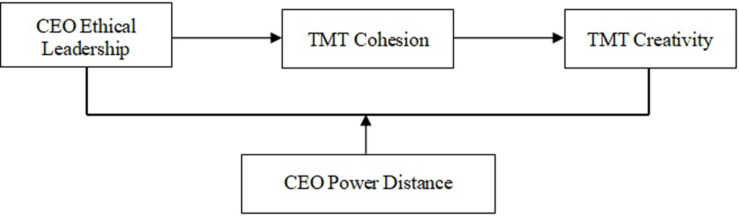
Conceptual model and hypotheses. TMT, top management team.

Practitioners in management rarely recognize the positive relationship between CEO ethical leadership and team creativity. This raises the issue of whether there is any boundary to such effect. To answer this question, the CEO’s personal values, especially cultural values, have to be taken into consideration (Miles et al., 1978; Bluedorn and Lundgren, 1993). Despite a considerable body of research on team creativity, it remains unclear whether CEO cultural values have moderating effects on the relationship between CEO ethical leadership and team creativity. Research suggests that power distance is the most theoretically relevant cultural value that may moderate the relationship between CEO leadership style and TMT creativity, particularly for firms in East Asia (e.g., Miles et al., 1978; Bluedorn and Lundgren, 1993). Therefore, this study tests the effect of CEO cultural values on the relationship between CEO ethical leadership and TMT creativity by focusing on the effect of CEO power distance, defined as the extent to which an individual accepts the unequal distribution of power in institutions and organizations (Kirkman et al., 2009). In sum, we propose a hypothetical model, as shown in Figure 1.

Considering that most of the current studies in this field provide evidence from western countries, our study uses data from China to provide empirical evidence from a different cultural context. Some leaders in China still hold the traditional view that there is an insurmountable gap between leaders and subordinates, with high power distance. Just as Bass (1985, p. 154) noted, in China, “leadership patterns. are still influenced by Confucian precepts.” However, with ongoing growth, many leaders in China have begun to change their traditional ideas and believe that leaders and subordinates can work together in a more equal way, with low power distance. Both situations exist now in China, giving us valuable opportunities to explore the impact of leader value orientation. In addition, compared with western countries, leaders in China are expected to set a moral model for their followers (Bass, 1998; Fu et al., 2010). Ethical leadership in China is often more highly esteemed, and employees in organizations are more likely to accept leaders who follow ethical norms. Therefore, ethical leadership may play a more pivotal role in motivating high performance in the Chinese context.

## Literature Review and Hypotheses

### CEO Ethical Leadership and TMT Creativity

CEO leadership style has a disproportionate, sometimes nearly dominating influence on TMT characteristics and outputs (Hambrick, 1994; Gong and Li, 2019; Tang et al., 2019). Recent research highlights important contingency factors, such as team ethical climate, related to the functioning of ethical leadership in teams (Lin et al., 2017). According to the definition of ethical leadership previously described (Brown et al., 2005), there are two components of an ethical leader, as a moral person characterized by attributes, such as honesty and fairness, integrity, trustworthiness, altruistic motivation, and justice (e.g., Treviño et al., 2003; Brown et al., 2005; Brown and Treviño, 2006), and as a moral manager who is expected to influence his or her followers’ ethical and unethical behavior through proactive efforts, such as actively communicating about ethics, setting high ethical standards, and using a reward system to ensure that those standards are followed (e.g., Treviño et al., 2003; Brown et al., 2005; Brown and Treviño, 2006).

The three dimensions of ethical leadership are morality and fairness, role clarification, and power sharing (Brown et al., 2005; Hoogh and Hartog, 2008), which are all motivational factors of TMT creativity (Hoogh and Hartog, 2008). First, morality and fairness represents a CEOs’ fair and moral behavior (Brown et al., 2005; Hoogh and Hartog, 2008). Ethical CEOs generally perform in an honest, trustworthy, fair, principled, and altruistic manner (Brown and Treviño, 2006; Hoogh and Hartog, 2008; Loi et al., 2012). Therefore, TMT members are more likely to feel psychologically safe to voice their new ideas and share their knowledge (Janssen, 2000, 2003; Loi et al., 2012), thus facilitating TMT creativity (West and Richter, 2008). Second, role clarification reflects ethical CEOs’ transparency, engagement in communication characterized by trust, openness and sincerity, promoting and rewarding ethical behaviors, and clarification of expectations and responsibilities (Brown et al., 2005; Hoogh and Hartog, 2008; Gong, 2018). Ethical CEOs advocate two-way open communication, listen sincerely to TMT members with patience, and encourage them to express their own opinions, thus stimulating TMT members to come up with novel ideas (Martins and Terblanche, 2003). Third, ethical CEOs’ power sharing includes advocating that TMT members participate in decision making and listening to other TMT members’ ideas and concerns (Brown et al., 2005; Hoogh and Hartog, 2008). Ethical CEOs offer a higher level of autonomy and freedom in TMT members’ work (Brown et al., 2005; Hoogh and Hartog, 2008; Oke et al., 2009; Tu and Lu, 2013), which fosters creativity (Amabile, 1997). TMT members have more control (Piccolo et al., 2010) and less constraints in their work, which contributes to proposing, promoting, and implementing new ideas (Tu and Lu, 2013). Moreover, TMT members led by an ethical CEO may be more willing to allow others to express their opinions, show respect, and consider others’ needs (Mayer et al., 2012). Consequently, TMT members are more likely to suggest creative ideas rather than withhold ideas to avoid possible intra-team tension. Thus, for these reasons, we expect a positive relationship between CEO ethical leadership and TMT creativity.

Hypothesis 1: CEO ethical leadership is positively related to TMT creativity.

### The Mediating Role of TMT Cohesion

Top management team cohesion refers to the extent to which TMT members are attracted to each other (Shaw, 1981; Kidwell et al., 1997). It reflects TMT members’ attachment to the team and synergistic interactions among them, including positive communication, conflict resolution, effective workload sharing, and collaboration with others on the basis of affective commitments, representing an emotional and motivational state among TMT members (Barrick et al., 1998; Shin and Choi, 2010; Tang et al., 2020). Wei and Wu (2013) consider it one of the most critical aspects influencing the TMT process, but team cohesion in TMTs has been little studied. In addition, although scholars have found that team cohesion influences team productivity (Mullen and Copper, 1994), team performance, and shared cognition (Carron et al., 2005), its relationship with ethical leadership is unclear. Stogdill (1974) and Tjosvold (1984) have found that team leaders who are people-oriented tend to enhance interpersonal cohesiveness. Michalisin et al. (2004) and Buyl et al. (2011) have suggested that team cohesion can be established or shaped by the team leader. Therefore, it is reasonable to expect that CEO ethical leadership has a positive influence on TMT cohesion.

First, ethical CEOs are honest, trustworthy, fair, principled and altruistic (Brown and Treviño, 2006; Hoogh and Hartog, 2008; Loi et al., 2012), and high in personal warmth toward others. From the perspective of social learning, because ethical CEOs set high ethical standards and promote and reward ethical behaviors, TMT members are more likely to behave in an ethical manner. According to this logic, TMT members led by an ethical CEO are more likely to behave ethically toward other members, avoid personal attacks on others, show respect, and consider others’ needs (Mayer et al., 2012), thus facilitating the formation of satisfaction and trust among members, which induces interpersonal attachment and liking among members. In addition, ethical CEOs are transparent and engage in two-way communication through trust, openness, and sincerity (Hoogh and Hartog, 2008). TMT members led by an ethical CEO may be more willing to allow others to express their opinions (Mayer et al., 2012), cooperate, and share critical information to reach a team consensus (Stogdill, 1974; Tjosvold, 1984; Peterson et al., 2003). As a result, TMT members are more likely to build strong internal relationships and experience positive interactions among members, thus becoming more cohesive (Barrick et al., 1998; Aubke et al., 2014). Second, ethical CEOs advocate for TMT members to participate in decision making. Through participating in decision making, TMT members are more likely to perceive that the TMT’s values and goals are consistent with the firm’s values and goals, and they will develop a collective sense that the TMT is legitimate and significant, which may induce TMT cohesion (Shin and Choi, 2010). Taken together, CEO ethical leadership may have a positive influence on TMT cohesion.

Scholars have indicated that a diverse and cohesive team becomes more creative (e.g., Van der Vegt and Bunderson, 2005; Shin and Zhou, 2007). Prior research has suggested that cohesive teams enjoy three advantages: enforceability of sanctions and norms, trust and reciprocity, and knowledge sharing (Coleman, 1988; Uzzi, 1996; Nahapiet and Ghoshal, 1998; Guler and Nerkar, 2012). TMT cohesion can affect TMT creativity through three ways. First, TMT cohesion encourages cooperation between members of the network, and the information about a member’s opportunistic behavior travels easily through the network, thus influencing his or her reputation (Coleman, 1988; Guler and Nerkar, 2012). Therefore, TMT cohesion reduces opportunistic behavior and encourages each team member to share valuable information and ideas (Guler and Nerkar, 2012), thus facilitating TMT creativity. Second, a cohesive TMT usually has a higher level of trust (Homan et al., 2007), which increases members’ investment of time, energy, and effort in sharing knowledge with others (Reagans and McEvily, 2003). In addition, a cohesive TMT is more tolerant of disagreement and dissent (Ensley et al., 2002) and more open to cooperation (Uzzi, 1996), thus leading to a culture of sharing and cooperating (Guler and Nerkar, 2012). Third, TMT cohesion can enhance communication and information sharing among team members (Staples and Webster, 2008; Tang et al., 2016, 2020), which is critical to team creativity (Gong et al., 2013; Tang et al., 2016). Accordingly, TMT cohesion, which can be generated through CEO ethical leadership, may facilitate TMT creativity. Hence, we propose:

Hypothesis 2: TMT cohesion mediates the relationship between CEO ethical leadership and TMT creativity.

### The Moderating Role of Power Distance

To have a comprehensive understanding of the relationship between CEO leadership and TMT creativity, the influence of the CEO’s cultural values must be considered (Heskett and Kotter, 1992; Bluedorn and Lundgren, 1993; Tang et al., 2015; Gong et al., 2019). Ethical leadership embraces positive behaviors of leaders, including serving as a role model, planning and setting appropriate goals, showing trust, support and concern, and communicating well with team members (Amabile, 1997; Amabile et al., 2004). It is important to note that, under the social learning process, followers not only observe leaders’ external behavior but also infer the beliefs and values that underlie their behaviors by listening to their words and observing their non-verbal behaviors and other social cues (O’Reilly and Pfeffer, 2000). The value orientation of leadership is particularly relevant in considering the influences of ethical leadership. Research has shown that power distance is the most theoretically relevant cultural value that may moderate the relationship between CEO leadership style and TMT creativity (e.g., Miles et al., 1978; Bluedorn and Lundgren, 1993). With a high degree of power distance, team members may not be willing to share information and knowledge candidly and openly (Bluedorn and Lundgren, 1993). Thus, power distance may weaken the positive relationship between CEO ethical leadership and TMT creativity.

Hofstede (1997) defines power distance as the extent to which the less powerful members of an organization or institution (such as the family) expect and accept power being distributed unequally in that organization or institution. Measurement of this cultural value actually suggests the degree to which inequality in information control is endorsed by followers as much as by leaders. In their study of the effects of power distance on firm business strategy, Bluedorn and Lundgren (1993) found a high degree of power distance to be more compatible with the so-called defender strategy, which concentrates on protecting current markets and serving current customers (Miles et al., 1978). In contrast, a low degree of power distance is more congruent with the so-called prospector strategy, which stresses risk-taking, flexibility, and aggressively searching for new products, new markets, and new growth opportunities (Miles et al., 1978). High-power-distance CEOs are generally characterized by centralized authority, a paternalistic management style, and so on. All of these characteristics are commonly observed in firms in China and other East Asian countries (e.g., Hofstede, 1997). This cultural value will arguably offset the positive effect of CEO ethical leadership on TMT creativity because of power inequalities among TMT members.

It has been found that TMTs with a high degree of power distance are not willing to discuss issues candidly and openly (Eisenhardt and Bourgeois, 1988). According to previous research on negotiation research, high power distance between leaders and members can impede the creative processes and activities in teams (e.g., Mannix and Neale, 1993). Highly power distant CEOs often believe that they can solve some problems correctly and forcefully, which can suppress other members’ creative problem solving capability. Moreover, feeling greater distance from the CEO, other members may not be willing to cooperate with others to deal with difficult or challenging tasks (Edmondson et al., 2003). When a group or team has a high level of power distance, the members of that group or team may have less motivation to share information and knowledge equally (Edmondson et al., 2003). More importantly, even when information is available, it is likely to be less effective in boosting team creativity because followers may prefer to wait for orders or decisions from leaders. In such cases, it may be unlikely that CEO ethical leadership exerts more significant effects on TMT creativity, so the positive relationship of CEO ethical leadership with team creativity may be particularly acute in TMTs led by high-power-distance CEOs. Accordingly, we predict the following:

Hypothesis 3: Power distance negatively moderates the relationship between CEO ethical leadership and TMT creativity. Other conditions being equal, the greater the power distance, the weaker the positive relationship between CEO ethical leadership and TMT creativity.

## Materials and Methods

### Sample and Methods

Given that the hypotheses would be tested at the TMT level, we collected data from the CEOs and several other top managers of companies located in mainland China. With the help of MBA students, we randomly selected companies across China in various industries and then sent an invitation letter to each firm’s CEO to illustrate the research aiming to understand the role of leadership and TMT creativity in the workplace. We guaranteed that the responses would be kept confidential and anonymous and only used for research purposes and sought further cooperation. To encourage participation, we told participants that they have right to retrieve and/or withdraw their information from the study at any time and promised that we would offer the findings of the study to each participating firm. With these guarantees, we received consent from 150 CEOs. Questionnaires were then administered to the CEOs and TMTs. The questionnaires were coded before distribution to match the several responses from each firm. Initially, research assistants delivered the questionnaires to the 150 companies. TMT members were invited to provide their perception of the CEO’s ethical leadership and team cohesion. Three months later, we mailed the surveys to these companies, and the CEOs returned the completed surveys. We asked the CEOs to report the TMT’s creativity; CEO power distance; their demographic information such as age, tenure, and education; and firm characteristics such as firm age, firm size, and ownership type. Finally we received 91 sets valid answers at a response rate of 60.1%. Of the final sample of 91 companies, 30 (32.9%) are state-owned enterprises, and 61 (67.1%) are non-state-owned enterprises such as foreign-invested enterprises and private firms. These firms are located all over China and in various industries: 56.1% of the sample are manufacturing firms, and the remaining 43.9% are non-manufacturing firms.

### Measures

Following the commonly used back-translation procedure, the scales were translated from English into Chinese and then back-translated into English by two independent bilingual individuals to ensure equivalency of meaning (Brislin, 1980). First, the measures were translated into Chinese by two researchers. Then, the translated measures were back-translated by another researcher. Finally, we checked to ensure that the final translated version matched the original English version.

#### CEO Ethical Leadership

Using the 10-item Ethical Leadership Scale (ELS) developed by Brown et al. (2005), non-CEO top team managers (including the chief finance officer and chief human resources officer) responded using a five-point response scale ranging from one (strongly disagree) to five (strongly agree). Sample items are “Our boss has the best interests of employees in mind,” “Our boss always makes fair and balanced decisions,” “Our boss always disciplines employees who violate ethical standards,” and “Our boss always defines success not just by results but also the way that they are obtained.” Top managers’ ratings of CEO ethical leadership were aggregated to the TMT level of analysis. Cronbach’s reliability coefficient alpha was 0.92, indicating acceptable reliability.

#### TMT Cohesion

Lee and Farh’s (2004) five-item scale was used to measure team cohesion. Non-CEO top team managers were asked to assess their perceived team cohesion. Sample items include “The top management team members get along with each other” and “The top management team members stick together.” Top managers’ ratings of TMT cohesion were aggregated to the TMT level of analysis. Cronbach’s reliability coefficient alpha was 0.84, indicating acceptable reliability.

#### TMT Creativity

A three-item scale developed by Farh et al. (2010) was used to measure TMT creativity. CEOs responded using a five-point response scale ranging from one (strongly disagree) to five (strongly agree). A sample item is “Our team output demonstrates that the team is capable of using existing information or resources creatively.” Cronbach’s reliability coefficient alpha was 0.89, indicating acceptable reliability.

#### Power Distance

Power distance was measured by a six-item scale from Erez and Earley (1987). CEOs evaluated the extent of power inequality between themselves and their subordinates, based on a five-point Likert scale, ranging from one (very low extent) to five (very high extent). Sample items include “It is necessary to use power to deal with many problems” and “Employees should not disagree with their leaders’ decisions.” Cronbach’s reliability coefficient alpha was 0.81, indicating acceptable reliability.

#### Control Variables

Similar to prior research, several control variables were controlled. At the individual level, following other researchers (e.g., Amabile, 1997; Shin and Zhou, 2007), CEO age, tenure, and education were controlled. Then, firm age, firm size, firm site, industry type, and the team’s cognitive diversity were controlled because these could affect individual team member creativity (Shin et al., 2012). The team’s cognitive diversity was measured using Van der Vegt and Janssen’s (2003) four-item measure. A sample item is “Team members and I have differences in the way of thinking.” Cronbach’s reliability coefficient alpha was 0.85, indicating acceptable reliability.

### Analytic Strategies

Data analysis was conducted in three phases. First, confirmatory factor analyses (CFAs) were conducted to test the validity of constructs. Specifically, the composite reliability of measurements was evaluated based on the value of composite reliability (CR) and average variance extracted (AVE), and the discriminant validity of measures was assessed by comparing the measurement model with competing models based on the comparisons of the fit indexes. Second, descriptive statistics and correlation analysis were calculated to understand the interrelations among the study variables. Finally, multiple regression analyses were used to test the hypotheses shown in Figure 1. Multiple regression analysis is widely used in management research due to its capability to estimate the influence of two or more variables on dependent variables at the same time. The multistep regression approach proposed by Baron and Kenny (1986) was used to figure out if there is a mediating effect.

MPLUS 7.0 was used for the CFAs to evaluate the discriminant validity of key variables, and SPSS 22.0 was used to calculate the descriptive statistics, correlation between variables, and multiple regression analyses.

### Ethics Approval Statement

Data in the study were voluntarily reported, and all participants were provided sufficient information to be able to give informed consent to participate in this study. Research respondents were guaranteed anonymity, were informed that the data would only be used for academic study, and were assured of the confidentiality of their data.

## Results

### Confirmatory Factor Analysis

Before testing the hypotheses, a series of CFAs were run to test whether the hypothesized model captured distinct constructs. In testing the measurement model for convergent validity, this study assessed the factor loadings, CR, and AVE. All the item loadings exceeded the suggested value of 0.6, and CR and AVE values also exceeded the recommended values of 0.7 and 0.5 in Table 2, respectively (Fornell and Larcker, 1981). The result of comparing the measurement model with four competing models is described in Table 1 and is consistent with many studies in the work team (unit) literature (Wei and Wu, 2013). To achieve an optimal ratio of sample size to the number of estimated parameters, the scale items were combined with the highest and lowest loadings by averaging them (Aryee et al., 2007) into three parcels for each variable following previous research (Zhang et al., 2012). The goodness-of-fit measure of the model was assessed using the χ^2^/*df*, comparative fit index (CFI), Tucker–Lewis index (TLI), root mean square error of approximation (RMSEA), and standardized root mean square residual (SRMR) with acceptable thresholds of >0.90, >0.90, <0.08, and <0.05, respectively (Schermelleh-Engel et al., 2003). Table 1 shows that the hypothesized four-factor model fits the data considerably better than any of the alternative models based on the comparisons of the fit indexes, with χ^2^ = 65.019, *df* = 48, CFI = 0.976, TLI = 0.966, RMSEA = 0.062, and SRMR = 0.049. After examining the alternative three-, two-, and one-factor models, the model comparison results in Table 1 show that a four-factor model fits the data considerably better than any alternative. Therefore, the discriminant validity of the constructs was confirmed. In addition, all of the factor loadings were significant, indicating convergent validity.

**TABLE 1:** Results of confirmatory factor analyses.

Model	χ^2^	df	CFI	TLI	RMSEA	SRMR
Four-factor model^a^	65.019	48	0.976	0.966	0.062	0.049
Three-factor model 1^b^	96.700	51	0.934	0.915	0.099	0.063
Three-factor model 2^c^	283.37	51	0.666	0.568	0.224	0.269
Two-factor model^d^	314.572	53	0.624	0.532	0.233	0.273
One-factor model^e^	279.829	54	0.675	0.603	0.214	0.145

*N = 91; CFI, comparative fit index; TLI, Tucker–Lewis index; RMSEA, root mean square error of approximation; SRMR, standardized root mean square residual. ^*a*^CEO ethical leadership, top management team (TMT) cohesion, TMT creativity, and power distance load on their respective factors. ^*b*^CEO ethical leadership and TMT cohesion load on one factor; TMT creativity and power distance load on their respective factors. ^*c*^CEO ethical leadership and TMT cohesion load on their respective factors; TMT creativity and power distance load on one factor. ^*d*^CEO ethical leadership and TMT load on one factor, and TMT creativity and power distance load on a second factor. ^*e*^All indicators load on one single factor.*

### Descriptive Statistics

The means, standard deviations, and bivariate correlations of the variables, including control variables, CEO ethical leadership, TMT cohesion, TMT creativity and power distance, are presented in Table 2. As expected, CEO ethical leadership is positively related to TMT cohesion (*r* = 0.66, *p* < 0.001) and TMT creativity (*r* = 0.67, *p* < 0.001). There is a positive correlation between TMT cohesion and TMT creativity (*r* = 0.60, *p* < 0.001). These results were consistent with the expected direction and provided preliminary data to confirm the hypotheses.

**TABLE 2:** Means, standard deviations, and correlations.

Variables	1	2	3	4	5	6	7	8	9	10	11
(1) CEO age	**–**										
(2) CEO tenure	0.42***	**–**									
(3) CEO education	–0.01	0.28**	**–**								
(4) Firm size	0.16	0.45***	0.35**	**–**							
(5) Firm ownership	0.19	0.10	0.36**	0.35**	**–**						
(6) Firm age	–0.05	–0.05	0.25*	0.38***	0.33**	**–**					
(7) Cognitive diversity	0.11	0.19	0.13	0.31**	0.22*	–0.02	***(0.85)***				
(8) CEO ethical leadership	0.15	−0.24*	0.02	0.22	0.21*	–0.02	0.24*	***(0.92)***			
(9) TMT cohesion	0.13	0.11	–0.05	0.21	0.01	–0.03	0.18	0.66***	***(0.84)***		
(10) Power distance	0.08	0.13	0.09	–0.10	–0.17	–0.06	–0.10	–0.12	0.01	***(0.81)***	
(11) TMT creativity	0.19	0.13	0.05	0.21	0.05	–0.10	0.25*	0.67***	0.60***	0.15	***(0.89)***
**Mean**	41.80	11.41	2.13	2.25	0.30	11.44	3.65	3.95	3.98	3.00	3.83
**SD**	6.43	7.15	0.74	0.86	0.46	9.45	0.75	0.64	0.60	0.73	0.78
**CR**	–	–	–	–	–	–	–	0.95	0.88	0.86	0.93
**AVE**	–	–	–	–	–	–	–	0.65	0.61	0.51	0.81

N = 91; ***p < 0.001, **p < 0.01, *p < 0.05; CR, composite reliability; AVE, average variance extracted; The bold values indicate Cronbach’s alpha appears along the diagonal in parentheses.

### Hypotheses Testing

Results of the hypothesis tests are summarized in Table 3. First, the relationship between the independent variable (i.e., CEO ethical leadership) and dependent variable (i.e., TMT creativity) was significant (M4, β = 0.69, *p* < 0.001). Thus, Hypothesis 1 was verified. Second, as Table 3 shows, the relationship between the independent variable (i.e., CEO ethical leadership) and the mediating variable (i.e., TMT cohesion) was also significant (M2, β = 0.68, *p* < 0.001). In addition, TMT cohesion was positively related to TMT creativity (M5, β = 0.61, *p* < 0.001). After adding TMT cohesion to the regression equation, the direct effect from CEO ethical leadership to TMT creativity decreased but remained significant (M6, β = 0.48, *p* < 0.001). These findings show that TMT cohesion partially mediates the relationship between CEO ethical leadership and TMT creativity. Thus, Hypothesis 2 was supported.

**TABLE 3:** Results of hierarchical regression analyses of the mediating and moderating effects.

	TMT cohesion		TMT creativity				
	Model l	Model 2	Model 3	Model 4	Model 5	Model 6	Model 7
**Control variables**							
CEO age	–0.01	0.01	0.22	0.23*	0.22*	0.23*	0.17*
CEO tenure	0.05	–0.05	–0.12	−0.22*	–0.15	−0.20*	–0.26
CEO education	–0.06	–0.04	0.01	0.02	0.04	0.03	–0.04
Firm age	–0.05	0.04	–0.13	–0.03	–0.10	–0.05	–0.04
Firm size	0.21	0.09	0.28	0.16	0.15	0.13	0.22
Firm ownership	–0.10	–0.20	–0.02	–0.12	0.04	–0.06	–0.06
Cognitive diversity	0.14	0.03	0.15	0.05	0.07	0.04	0.08
**Independent variable**							
CEO ethical leadership		0.68***		0.69***		0.48***	0.71***
**Mediator**							
TMT cohesion					0.61***	0.31**	
**Moderator**							
Power distance							0.14
**Interaction**							
Ethical leadership × power distance							−0.25**
*R*^2^	0.07	0.48	0.16	0.57	0.51	0.62	0.66
Δ*R*^2^	0.07	0.40	0.16	0.41	0.35	0.46	0.05
*F*	0.72	7.11***	1.79	10.59***	8.18***	11.36***	11.87***
Δ*F*	0.72	48.14***	1.79	60.54***	44.38***	37.67***	8.99**

N = 91; ***p < 0.001, **p < 0.01, *p < 0.05.

**FIGURE 2:**
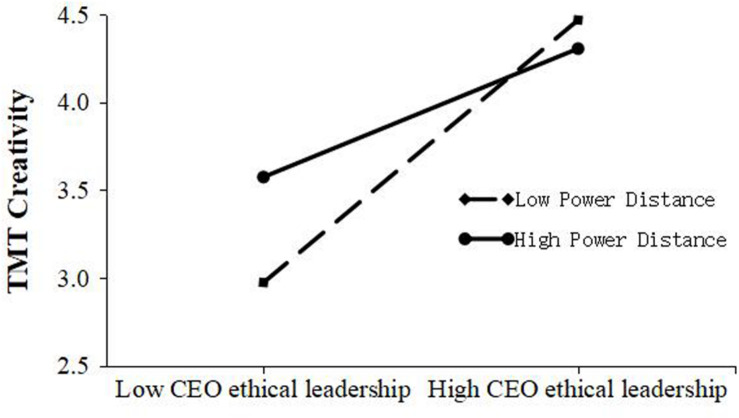
Simple moderating effect of power distance on the relationship between CEO ethical leadership and TMT creativity.

Table 3 shows that the interaction of CEO ethical leadership and power distance has a significant effect on TMT creativity (M7, β = −0.25, *p* < 0.01). Therefore, Hypothesis 3 was supported. Finally, moderating effect graphs were drawn based on one standard deviation above the mean and one standard deviation below the mean. Figure 2 shows that, compared with lower levels of power distance, the positive relationship between CEO ethical leadership and TMT creativity is weaker at a higher level of power distance.

### Supplementary Analyses

In order to further examine our model depicted in Figure 1, PROCESS analysis with 5,000 bootstrap samples was conducted to test our hypotheses. In Hypothesis 1, the relationship between CEO ethical leadership and TMT creativity was significant (*B* = 0.62, *SE* = 0.14, *p* < 0.001, 95% CI = [0.33, 0.89]). The indirect effect of TMT cohesion between CEO ethical leadership and TMT creativity was positive and significant (*B* = 0.27, *SE* = 0.13, *p* < 0.01, 95% CI = [0.07, 0.59]), supporting Hypothesis 2. The interaction between CEO ethical leadership and power distance was significant in predicting TMT creativity (*B* = −0.39, *SE* = 0.14, *p* < 0.01, 95% CI = [−0.67, −0.12]); thus, Hypothesis 3 was supported. The full moderated mediation model was tested by calculating the indirect effects of CEO ethical leadership on TMT creativity via TMT cohesion at high versus low levels of power distance, but all the confidence intervals about the mean, and one standard deviation above the mean and one standard deviation below the mean, do not include zero. This showed that the indirect effect of CEO ethical leadership on TMT creativity via TMT cohesion under high power distance was the same as that under low power distance, so the moderated mediation was not supported.

## Discussion

This study conceptualized and tested a model that provides insight into the linkages between CEO ethical leadership and TMT creativity. The results show that ethical leadership by the CEO is positively related to TMT creativity and that the relationship is mediated by team cohesion and negatively moderated by power distance. These outcomes offer several theoretical and managerial implications.

### Theoretical Implications

This study makes a number of related theoretical contributions in extending our knowledge on leadership and team creativity in general. First, it fills an important research gap in the research of ethical leadership by focusing on how CEO ethical leadership impacts team creativity at the TMT level. Most previous empirical studies have focused on the influence of ethical leadership on individual-level outcomes (Tang et al., 2015; Haller et al., 2018; Quade et al., 2019) but neglected the results of high-level outcomes. Adding to previous studies that ethical leadership is related to several positive outcomes (Brown et al., 2005; Hoogh and Hartog, 2008; Haller et al., 2018; Quade et al., 2019), this study adds the additional outcome of TMT creativity, which is significant given the growing need for innovative leaders and organizations (Moore and Wang, 2017). Given that higher-level leaders are different from those at lower levels in terms of their high position, responsibility, and potential to influence the organization (Carmeli and Schaubroeck, 2006; Ling et al., 2008; Moore and Wang, 2017), this study leads to a better understanding of the influence of CEO ethical leadership on the TMT. This study answers the call for more research on ethical leadership at higher levels and confirms the continuing need to learn more about developing this influential factor.

Second, this study not only reveals the mechanism of CEO ethical leadership on TMT creativity but also verifies the relationship with empirical data from Chinese firms, enriching the relevant research on the influencing factors of TMT creativity. Although some research already exists on the antecedents of TMT creativity (Shin and Zhou, 2007; Shin et al., 2012), little research has examined how TMT cohesion mediates the relationship between CEO leadership and TMT creativity. It confirms that CEOs’ behaviors can “trickle down” to TMT members (Mayer et al., 2009). TMT members could learn ethical behaviors through role modeling and vicarious learning (Bandura and Walters, 1977) and behave ethically toward other members (Mayer et al., 2012), thus facilitating cohesive actions among the TMT. As a result, TMT members establish a high-quality relationship with each other and feel psychologically and psychosocially safe (Yidong and Xinxin, 2013), which is necessary for TMT creativity.

Third, with the focus on the moderating role of CEO power distance, we respond to the previous suggestion that power distance can be seen as the most theoretically relevant cultural value that may moderate the relationship between CEO leadership style and TMT creativity, particularly for firms in East Asia (e.g., Miles et al., 1978; Bluedorn and Lundgren, 1993). Thus, this study provides an important finding toward establishing a more nuanced understanding of CEO cultural values which can be used to enhance team creativity. The introduction of power distance as a moderator deepens our understanding of the boundary of the relationship between CEO ethical leadership and TMT creativity. High power distance between leaders and team members can impede the creative processes in teams (Mannix and Neale, 1993), suppressing members’ motivation to solve problems and hindering members from sharing information and knowledge. Leaders unwilling to “get off their high horse” will greatly frustrate the enthusiasm of employees to make innovative contributions to the organization. Thus, more attention needs to be paid to the key influence of power distance in future research on creativity, especially in a culture of deep-rooted high power distance such as China. As there are few empirical studies, we call for the validation of the results in other countries.

### Managerial Implications

The findings from this research have practical implications as well. First, in a competitive environment, CEOs can improve the creativity of TMT members by adopting ethical leadership, giving members an open and equal atmosphere of communicating, learning, and sharing (Tang et al., 2016). CEOs can also encourage them to participate in decision making, to promote creativity and adapt to changes in the external environment. Second, the TMT usually has rich specific knowledge and excellent practical skills, which are critical in improving business efficiency. However, the transformation process of tacit knowledge is much more difficult than that of explicit knowledge. Therefore, the CEO should also pay attention to improving the cohesion of the TMT. It is necessary to strengthen the cohesion of the TMT so that the members can experience the identification of teamwork. This requires organizations not only to improve the knowledge and skill of team members but also to pay attention to the construction of team climate, which stimulates internal motivation (Lin et al., 2017; Tang et al., 2020). Third, in problem solving, leaders should have equal and fair communication with team members instead of imposing power orders. This is conducive to emotional bonding between team members, to increase mutual understanding and enhance knowledge sharing (Staples and Webster, 2008; Tang et al., 2016). Therefore, team members can make full use of their own advantages, give full play to their creative potential, and ultimately enhance the competitive advantage of the organization.

### Limitations and Future Research

There are some limitations in this study. First, the study is correlational, and the results cannot prove the causality implied in our research model. Thus, future research could use experimental methodology to support conclusions on causality. Second, although this study explores the impact of ethical leadership on TMT creativity, in-depth analysis is required on whether and how humble leadership, inclusive leadership, and other leadership types can impact TMT behavior. Finally, although this study confirms the mediating role of team cohesion between CEO ethical leadership and TMT creativity, future research can try to explain the mechanism between them from multiple perspectives. For example, future research could explore the underlying mechanism of CEO ethical leadership in TMT creativity through psychological empowerment, team behavioral integration, and other mediators.

## Data Availability Statement

The datasets generated for this study are available on request to the corresponding author.

## Ethics Statement

The studies involving human participants were reviewed and approved by School of Management, Shandong University. The patients/participants provided their written informed consent to participate in this study.

## Author Contributions

All authors listed have made a substantial, direct and intellectual contribution to the work, and approved it for publication.

## Conflict of Interest

The authors declare that the research was conducted in the absence of any commercial or financial relationships that could be construed as a potential conflict of interest.
